# Epigenetic program of ontogenesis and hyperfunction theory: reinterpreting the mechanisms of aging

**DOI:** 10.3389/fragi.2026.1735269

**Published:** 2026-01-21

**Authors:** Lev Salnikov

**Affiliations:** AntiCA Biomed, San Diego, CA, United States

**Keywords:** aging, epigenetic program, genome methylation, hyperfunction theory, ontogenesis

## Abstract

This paper presents a comparative analysis of the relationship between aging, the epigenetic program of ontogenesis, and the main postulates of the hyperfunction theory. The discussion highlights points of convergence between these frameworks and proposes a unified interpretation. According to the hyperfunction theory, aging arises from the continued activity of growth and regulatory pathways after reproductive maturity, as more cells shift from proliferation to functional maintenance while retaining high metabolic and signaling activity. However, this process does not represent a simple enhancement of specialized cellular functions. Instead, it reflects a redistribution of intracellular resources from self-sufficiency to the performance of specialized functions. Building on earlier findings on genome methylation dynamics, we argue that the epigenetic program of ontogenesis regulates primarily the genomic regions responsible for cell differentiation. This unbalanced regulation results in a gradual drift of the active epigenetic landscape toward maladaptation. Consequently, the hyperfunctional state observed during aging is not the primary cause but a downstream effect of this one-sided epigenetic influence. Thus, the main cause of aging is not software errors in old age, but the lack of feedback between the activity of domestic and specialized genes in the body’s cells. The approach presented in the article points to the promise of new approaches to rejuvenation based on restarting the epigenetic program of cells. This direction is aimed at restoring the balance of genomic activity underlying aging and offers potential measures to restore genomic balance.

## Introduction

The importance of understanding the mechanisms of aging cannot be overestimated, because only by identifying the basis of this phenomenon can we control it. By analyzing the data currently available, it is necessary to determine which of these data relate to the causes of aging and which to its consequences (De Magalhães, 2024). Recently, researchers in the field of biogerontology have focused their attention on studying the body’s development program and, instead of viewing aging exclusively as the result of the accumulation of molecular damage, they view it as a temporal mismatch or deviation of developing ontogenetic programs. Within the framework of the hyperfunction model, the cause of aging is considered to be the functioning of growth modules, leading to an exit beyond their adaptive window. It should be noted that the theory of hyperfunction itself can be defined as the principle according to which, during the development of an organism, an increasing number of its cells switch from a state of division to a state of rest with a constantly increasing performance of specialized functions. At the same time, available data from epigenetics, reprogramming biology, systems-level transcriptomics, and evolutionary concepts confirm this point of view, placing developmental program drift on a par with stochastic changes and classic signs of aging (Porter et al., 2021; Meyer and Schumacher, 2024). The presented approaches to the causes and mechanisms of aging leave open a very important question: what is the main cause of aging within the framework of these concepts—disruptions in the work of epigenetic mechanisms of ontogenesis program implementation (Yang et al., 2024) or disadaptive changes in metabolism arising from the implementation of the ontogenesis program lead to epigenetic disorders. This article presents a comparative analysis of our understanding of the basic mechanisms of aging from the point of view of the body’s implementation of the ontogenesis program. In our understanding, the ontogenesis program is a sequence of activation of genes necessary for the differentiation of all specialized cells of the organism. This program is implemented through chromatin modification, mainly through histone acetylating, as well as through the process of gene methylation (Lee et al., 2020). It is worth noting that the ontogenesis program essentially completes its work during the prenatal period, forming all the organs and tissues necessary for the body. Continuing to work in the postnatal period, the ontogenesis program is aimed at its main goal—the body reaching sexual maturity. Presenting our point of view on the causes and main mechanisms of aging, based on the implementation of the ontogenesis program, we will demonstrate its explanatory capabilities using the example of the theory of hyperfunction. The theory of hyperfunction was first presented by Dilman and Ward (1992) from the perspective of neuroendocrine regulation of the body and was later developed in the works of other authors. The authors of the theory of hyperfunction argue that aging is caused not by general, stochastically determined degradation and destruction of cellular structures, but by the continued activation of cellular functions. These functions were useful at an early age, but over time they become maladaptive and destructive. The theory of hyperfunction is based on the idea that the biological pathways that regulate growth and development, particularly those involved in cell proliferation, metabolism, and stress response, remain active longer than they should. In a young organism, these processes promote growth, repair, and efficient functioning. However, as the organism ages, the same pathways that once played a useful role remain active, leading to cell degradation and damage, inflammation, and age-related diseases (Blagosklonny, 2007a; Blagosklonny, 2007b; Gems, 2022; Blagosklonny, 2021; Blagosklonny, 2022; Blagosklonny, 2023). In biogerontology, the theory of hyperfunction gained prominence mainly in connection with experimental work on the use of calorie restriction (CR), although while this method has been shown to increase the lifespan of laboratory animals, attempts to use it in humans have not yielded the same results (Flanagan et al., 2020). It should be noted that the basic principle of the hyperfunction theory essentially repeats Williams’ views, as set out in his theory of pleiotropy (Williams, 1957). The main drawback of the hyperfunction theory is the lack of explanation for the reason for the preservation of a constant increase in specialized functions in the body’s cells, and especially the fact that no direct experimental confirmation of this has been obtained. The available data only indicate an increase in the energy consumption of these functions (Amorim et al., 2022). Our view of the main causes and mechanisms of aging is based on understanding them as a by-product of the implementation of the ontogenesis program (Salnikov, 2022). In the following, we will compare and contrast our understanding of the relationship between aging and ontogenesis with the main statement of the hyperfunction theory, focusing on where they coincide or diverge. This article uses a comparative analytical model to evaluate the relationship between the epigenetic program of ontogenesis and the theory of hyperfunction of aging. The comparison is based on four criteria: causal primacy, level of biological regulation, temporal origin, and reversibility of age-related changes.

Regarding causal priority, the hyperfunction theory interprets aging as the result of the continued activity of signaling pathways associated with growth and metabolism beyond their adaptive developmental window. In contrast, the ontogenetic epigenetic model gives causal priority to the asymmetric regulation of the cellular genome during development, in which the part of the genome associated with differentiation remains predominantly active compared to the part of the genome responsible for maintaining vital functions.

In terms of the level of biological regulation, the hyperfunction theory operates mainly at the level of intracellular signaling pathways (e.g., insulin/IGF-1 and mTOR), whereas the model proposed here localizes the origin of aging at the level of genome-wide epigenetics. From this point of view, the hyper activation of signaling pathways is a downstream manifestation of a permanent shift in transcription and translation that is established during ontogenesis.

As for the temporal origin, the hyperfunction theory emphasizes the dysregulation that occurs after reproductive maturity. In contrast, the ontogenetic epigenetic model postulates that the foundations of aging are laid earlier, during development and maturation, as a consequence of the long-term implementation of a differentiation program that continues to shape the allocation of cellular resources throughout adulthood.

Finally, with regard to reversibility, the hyperfunction theory suggests, that aging can be influenced by suppressing certain signaling pathways. The model we use suggests that effective rejuvenation requires the restoration of genomic balance between maintenance and specialized gene activity, especially in post-mitotic cells. It follows that pathway inhibition alone may not be sufficient to reverse age-related functional decline.

## Implementation of the ontogenesis program and signs of hyperfunction at the level of mRNA synthesis and translation control

For a deep understanding of the main causes of aging and its mechanisms, a multicellular organism, where these phenomena occur, should be considered not in isolation, but in terms of its role in the population and the purpose of its own ontogenesis program. Representatives of the kingdom of single-celled organisms in the biosphere achieve their main goal, self-preservation, through cell division, where only those that have passed natural selection survive. In multicellular organisms, this task is accomplished through reproduction, also preserving species that have passed this barrier. Thus, from the point of view of population self-preservation, the main moment in the ontogenesis of any multicellular organism is the period of its fertility. Let us dwell on this point in more detail. The fundamental cause of aging is a decrease in reparative capabilities at the cellular level (Kenyon and Gerson, 2007; Moskalev et al., 2013; Guo et al., 2022). In our work, we have presented our point of view on this mechanism. We distinguish two functional parts in the genome of an organism: home genes (HG), which are responsible for the processes necessary for any cell, and integrative genes (IntG), which allow cells to perform all the specialized functions of the organism (Wei and Ma, 2017; Salnikov and Baramiya, 2021). This division of the cellular genome is confirmed by the analysis of age-related changes in the level of methylation in these gene groups, demonstrating a very large difference between them. We conducted a meta-analysis of human genome methylation data from an open source EWAS data hub (https://ngdc.cncb.ac.cn/ewas/datahub). We analyzed 100 genes, which were divided into two functional groups: HG, responsible for maintaining vital functions, and integrative genes IntG. Significant differences in absolute methylation levels in the methylation of promoters were found between the HG and IntG groups (p < 0.0001, t-test). In addition, genes belonging to the IntG group showed a reliable decrease in methylation with age, while HG levels remained constant (Salnikov et al., 2022). Although biological clocks based on genome methylation and transcriptomics clocks may be accurate, they are mechanistically agnostic. This means that age prediction alone does not allow us to distinguish between programmed ontogenesis and accumulated variability. Mathematical modeling shows that clocks can arise exclusively from stochastic variations, even in response to interventions such as CR and reprogramming, which cautions against over interpreting clocks as direct indications of a developmental “program” (Porter et al., 2021). By studying the age-related dynamics of the mRNA levels of these gene groups, we obtained results showing a gradual decrease in the HG gene group and a simultaneous increase in the IntG gene group. The results indicate a gradual shift from the high mRNA levels of HG genes observed at an early age to an increase in the mRNA levels of the IntG gene group observed from the beginning of the fertility period. At the same time, the trend of increasing IntG gene levels continued until the end of the observation period (Salnikov et al., 2023; Salnikov et al., 2024b). These data are direct confirmation of the redistribution of gene RNA synthesis in favor of the group responsible for specialized functions of the body’s cells, confirming the fact of a constant increase in functions or the main idea of the theory of hyperfunction. Indeed, it can be argued that this trend is entirely justified from the point of view of species survival, bringing the organism to its most competitive form during the critical period of fertility, demonstrating pleiotropy properties for the IntG gene group. Thus, from the data presented, we see that at the level of RNA production of the cellular genome, there is indeed a “hyperfunction effect,” expressed in a gradual increase in the number of mRNAs of specialized genes of the cellular genome. However, while agreeing with the main statement of the hyperfunction theory that we have established, although we obtain an answer to the question “why” and what goal natural selection pursued in preserving this property of the multicellular cell genome, we do not obtain an answer to the question of what the mechanism of its implementation is. Examining the processes of protein translation in the cells of the body and its changes with age will help us to gain a deeper understanding of the mechanisms of hyperfunction and their connection with the process of ontogenesis. When analyzing the amount of mRNA in cells, it is necessary to take into account not only the activity of translated genes, but also the stability of the mRNA itself, which is associated with the process of their translation and ultimately, in the event of a change in their stability, affects the amount of these molecules in the cell. According to a number of authors, a more intensive and efficient translation process usually increases mRNA stability, while faster and smoother elongation (codon optimization) stabilizes these molecules, and suppression or termination of translation destabilizes them (Ingolia et al., 2009; Fabian et al., 2010; Wu and Bazzini, 2023). Ribosome chains called polysomes, which can be “free,” or located in the cytoplasm carry out all protein synthesis in cells, or “bound,” attached to the cell membrane. This division of polysomes is related to the function of the proteins they produce. Proteins necessary for the cells own existence and encoded by home genes are produced on free polysomes, while proteins responsible for specialized cell functions are synthesized on membrane-bound polysomes represented in cells by their endoplasmic reticulum (ER) (Lashkevich and Dmitriev, 2021; Carter et al., 2020; Bose et al., 2020). This distribution of polysomes is entirely justified, since virtually all proteins that provide specialized functions of differentiated cells in the body are associated with its membrane structures. This connection ensures both external and internal secretion of specialized proteins in these cells. Total protein production peaks in early adulthood, followed by an overall decline (Kim and Pickering, 2023). It can be argued that this decline affects proteins that serve the cellular infrastructure to a greater extent. Apparently, these changes reflect the stages of implementation of the epigenetic program of ontogenesis, and in the case of an increase in its duration, changes in the ratio of free and EPR-bound polysomes occur more slowly. As an example, data obtained for this indicator in *Heterocephalus glaber* show that the amount of polysomes-bound proteins in its liver cells is significantly lower than in *Mus musculus*, and this ratio of free and polysomes-bound proteins in *H. glaber* cells is maintained throughout its life, possibly being one of the factors of its longevity (Vays et al., 2022). In addition, some studies suggest that with age, the number or area of ER within differentiated cells increases (Das, 2021), increasing the number of “seats” for bound polysomes. Aging and chronic stress often lead to rare dividing and post-mitotic cells demonstrating an increase in the number and surface area of certain intracellular membrane systems. Constant biosynthesis and proteostatic demand, regulated by contact sites, organelle biogenesis, and membrane lipid remodeling occur against a backdrop of reduced degradation flux, contributing to net accumulation and sometimes remodeling of membranes in long-lived cells (Gemmer et al., 2023). Summarizing the data presented, we can conclude that there is a tendency for the synthesis of proteins responsible for performing specialized functions in cells to increase with age, accompanied by a decrease in the synthesis of proteins responsible for maintaining cellular infrastructure. In slowly dividing and post-mitotic cells, which form the basis of most tissues in the body, the switch to specialized protein production occurs rather slowly, especially in post-mitotic cells, whose lifespan is comparable to that of the organism itself. As a result, it is in these cells that the redistribution of resources becomes of leading importance, since their physical replacement through the process of division is impossible. In addition, it is in these cells that the rate of protein synthesis and metabolism first increases with age and then begins to decline due to the depletion of their own resource base (Stein, et al., 2022; Kitada and Koya, 2021; Lashkevich and Dmitriev, 2021; Lanz et al., 2022). Often, such processes in the body with age lead to the accumulation of so-called senescent cells, and many scientific studies have been devoted to investigating their role in the processes of aging and the development of age-related diseases (Moiseeva et al., 2022; Marcozzi et al., 2022; Wagner and Wagner, 2023; Roger et al., 2021; Öztürk et al., 2020; Merulla et al., 2013; Rajendran et al., 2019; Kotani et al., 2023; Farrell and Ryan, 2020; Pluquet et al., 2015; Wang et al., 2021; Gil-Hernández and Silva-Palacios, 2020; Bose et al., 2020). Summarizing the presented data, we can say that both at the level of RNA production and at the level of protein translation, we observe an increase in the production of specialized proteins, but we can only observe an increase in the functions themselves from the onset to the end of the organism’s fertility period. After its completion, we observe not an increase in the specialized functions themselves, but an increase in the costs of their execution against the background of a decrease in the rate of synthesis and exchange of cellular proteins in general (Salnikov et al., 2024a).

## Discussion

Proponents of the hyperfunction theory argue that aging is a consequence of the body’s inability to shut down regulatory pathways that stimulate cells to perform their specialized functions when they are no longer needed. According to the authors, this phenomenon is essentially caused by the insulin/IGF-1 and mTOR signaling pathways present in cells, which regulate cell growth and metabolism, switching them from a state of division and growth to a state of rest (Leontieva et al., 2015). In the context of aging, the constant activation of such pathways leads to accelerated cell damage, decreased resistance, and increased susceptibility to age-related diseases (Leontieva and Blagosklonny, 2016). To stop the aging process from the point of view of hyperfunction theory, one should simply stop this phenomenon itself. To achieve an anti-aging effect, interventions that inhibit these pathways are proposed, including calorie restriction (Wang et al., 2024; Ealey et al., 2024; Wei et al., 2024) and the use of rapamycin (Pelton, 2022; Sabini et al., 2023), which, although they produce certain results in experimental animals, apparently cannot radically affect their lifespan. It should be noted here, that hyperfunction alone cannot be the main cause of aging, since it implies the presence of a sustained regulatory shift, the origin of which lies beyond the pathway-level control. From our point of view, the phenomenon of hyperfunction itself is not the cause of aging, but a consequence of the implementation of the epigenetic program of multicellular development, which, as we noted earlier, is aimed at achieving the maximum potential of the organism during its fertile period (Bueno Juan and Ángel, 2014). At the same time, the gradual shift of cellular resources to perform specialized functions is due to the lack of a direct connection between the closed system of regulation of specialized genes at the organism level, represented by IntG, and the internal regulation of the activity of housekeeping genes HG (Salnikov and Baramiya, 2020). From the perspective of our proposed concept, aging arises as a result of specific causes and their consequences, rather than as a result of independent events caused by regulatory disruption in later life. The epigenetic program of ontogenesis predominantly supports transcriptional activity in genomic regions responsible for cell differentiation, while the regulatory activity of gene networks responsible for maintaining vital functions remains relatively limited. This constant asymmetry leads to a progressive redistribution of transcriptional and translational resources in favor of specialized cellular functions. The phenomenon described by the hyperfunction theory arises as a phenotypic consequence of this redistribution, leading to increased metabolic demand and signaling activity in differentiated and post-mitotic cells. Aging occurs when the cumulative functional load exceeds the declining ability to maintain the cellular infrastructure, leading to a decrease in reparative potential, proteostatic imbalance, and functional deterioration at the tissue and organism levels. In this sequence, hyperfunction is not the root cause of aging, but a downstream manifestation of a long-term epigenetic bias established during ontogenesis. In addition, the functioning of a multicellular organism is based on the use of highly specialized, rarely dividing, and post-mitotic cells The cessation of their division processes enhances the redistribution of cellular resources with the participation of the ER, with some of them transitioning into senescent cells without the possibility of replenishing the cell pool through division, making this process irreversible. It is precisely the reliance of the organism as a whole on the creation and functioning of highly specialized post-mitotic cells, make them the “bottleneck,” the limiting factor that ultimately limits the lifespan of multicellular organisms. Thus, in order to control and stop the aging processes in multicellular organisms, it is necessary not only to restart the ontogenetic program in the cellular genome, but also to create a situation where the location of the postmitotic cell itself in the tissue structure is preserved. To date, there are a number of studies devoted to attempts to slow down aging (Lan et al., 2019; Sun et al., 2020; Fennel et al., 2024; Chen et al., 2024; Tang et al., 2024; Wan et al., 2024; Guo et al., 2022). One of the promising areas of scientific research aimed at stopping the aging process is attempting to achieve this result through cellular reprogramming (Zhang et al., 2020; Melo Pereira et al., 2019; Mahmoudi et al., 2019; Chen and Skutella, 2022; Chiavellini et al., 2021; Mitchell et al., 2024; Matteini et al., 2024; Simpson et al., 2021). Translational gerontology is investigating whether the reconfiguration of developmental programs through partial reprogramming, epigenetic stabilization, or modulation of nutrient perception can restore youthful functions without erasing cellular identity or creating oncogenic risk (Sahu et al., 2024; Wang et al., 2020). However, delivering the signaling proteins used in this method to most postmitotic cells remains an unresolved challenge. This work does not claim to provide direct experimental evidence of causal relationships at the level of lifespan and aging. The article aims to integrate transcriptomic, epigenetic, and evolutionary data into a mechanistically consistent explanatory model.

## Conceptual synthesis and testable implications

From our point of view, the main direction that could potentially overcome the ontogenetic and, consequently, age-related limitations imposed by evolution on multicellular organisms is a new direction in cellular rejuvenation, such as the cellular reprogramming and cellular autocloning (Salnikov, 2024a; Salnikov, 2024b). The proposed mechanism involves artificially stimulating cell nucleus division, resulting in the rapid destruction of one of the daughter nuclei, without the actual physical division of the cell. The goal of this process is cellular reprogramming with simultaneous renewal of the cell nucleus, ultimately producing a renewed clone. At the same time, this process also achieves renewal of the ER, but without physical division of the cell into daughter cells. This plays an important role in the process, allowing us to obtain a renewed post-mitotic cell in the highly differentiated tissue structure where it was located before the autocloning process began. Recent advances in the field of cell genome manipulation (Marti Gutierrez et al., 2025) give hope for a solution to this problem. In conclusion, we note that by comparing our understanding of the relationship between aging and ontogenesis with the main statement of the hyperfunction theory, we can focus on where they combined into one. The theory of hyperfunction is based on the gradual transition of an increasing number of cells in the body from a state of growth and division to a state of rest, while maintaining constant cellular regulation activity in this direction. Agreeing with the facts that point to this trend, we show that what is happening is not simply an increase in specialized functions, but a redistribution of intracellular activity and resources from infrastructure needs to the performance of functions required by the body. This situation leads to a gradual decrease in the overall level of protein synthesis and a decrease in the rate of their metabolism, observed over time, starting from the end of the fertility period. Considering the data on genome methylation presented earlier, it can be argued that the epigenetic program of ontogenesis regulates only the activity of that part of the genome that is responsible for cell differentiation. This one-sided influence leads to a drift towards maladaptation of the working epigenetic program. Thus, we can conclude that the phenomenon of hyperfunction itself is not the cause but the consequence of such a one-sided influence. The primary causes of aging are not deviations in the development program in the post-reproductive period or disturbances in the program itself, but the principle of organization of a multicellular organism based on the use of only a specialized part of the cellular genome. Approaching the analysis of aging from the perspective of the implementation of the epigenetic program of ontogenesis, developed by evolution on the basis of natural selection, allows us to understand which processes of aging are fundamental and which are simply their result. The ontogenetic approach shows directions that will eventually allow us to gain control over the processes of aging using new approaches, such as the cellular reprogramming and cellular autocloning.

## Data Availability

The original contributions presented in the study are included in the article/supplementary material, further inquiries can be directed to the corresponding author.
